# Critical Thickness of Free-Standing Nanothin Films Made of Melted Polyethylene Chains via Molecular Dynamics

**DOI:** 10.3390/polym13203515

**Published:** 2021-10-13

**Authors:** José Antonio González-Mijangos, Enrique Lima, Roberto Guerra-González, Fernando Iguazú Ramírez-Zavaleta, José Luis Rivera

**Affiliations:** 1 Facultad de Ciencias Físico-Matemáticas, Universidad Michoacana de San Nicolás de Hidalgo, Morelia 58000, Mexico; 2132223c@umich.mx (J.A.G.-M.); feramirez@umich.mx (F.I.R.-Z.); 2 Laboratorio de Fisicoquímica y Reactividad de Superficies (LaFReS), Instituto de Investigaciones en Materiales, Universidad Nacional Autónoma de Mexico, Circuito Exterior S/N, CU, Del. Coyoacán, Ciudad de Mexico 04510, Mexico; lima@iim.unam.mx; 3 Facultad de Ingeniería Química, Universidad Michoacana de San Nicolás de Hidalgo, Morelia 58000, Mexico; roberto.guerra@umich.mx

**Keywords:** critical thickness, polyethylene, molecular dynamics, radius of gyration, nanothin layers

## Abstract

The mechanical stability of nanothin free-standing films made of melted polyethylene chains was predicted via molecular dynamics simulations in the range of 373.15–673.15 K. The predicted critical thickness, $t_{c}$, increased with the square of the temperature, *T*, with additional chains needed as *T* increased. From *T =* 373.15 K up to the thermal limit of stability for polyethylene, $t_{c}$ values were in the range of nanothin thicknesses (3.42–5.63 nm), which approximately corresponds to 44–55 chains per 100 nm^2^. The density at the center of the layer and the interfacial properties studied (density profiles, interfacial thickness, and radius of gyration) showed independence from the film thickness at the same *T*. The polyethylene layer at its $t_{c}$ showed a lower melting *T* (<373.15 K) than bulk polyethylene.

## 1. Introduction

Polymeric membranes are a widespread separation technology used to solve many scientific and industrial problems. Commercial membranes used for gas separation are very thin, in the range of 100 to 500 nm, and the permeability to selectively separate gases increases as the thickness of the layer decreases [1,2,3]. Polyethylene membranes are permeated by gases with low critical temperatures independently of the pressure differential, while gases with high critical temperatures permeate the membranes in processes that are highly dependent on the pressure differential, which can indicate an affinity between small molecules and the polyethylene layer [4]. The solution–diffusion model is the most accepted transport mechanism of gases through a membrane [5,6], and it has three barriers: (1) adsorption of permeable gases at the polymer surface (commonly exposed to the permeable gas at a high pressure), (2) diffusion of the adsorbed gases in the polymer layer, and (3) desorption of permeated gases at the opposed interface (commonly exposed to the permeated bulk gas phase at a lower pressure). Therefore, if the thickness of the layer is decreased, the role of the second barrier is minimized.

Previous studies have shown that very thin layers of polymers [7] and atomistic systems [8] are mechanically unstable and break down into droplets below a certain critical thickness, $t_{c}$. Usually, the polymeric membranes are supported on porous substrates to enhance the mechanical stability of the system [9]. It is theorized that, in unsupported very-thin layers, attractive surface forces (disjoining pressures) increase the amplitude of capillary waves, causing the thin films to shatter, provided they are sufficiently thin. De Vries postulated that if the films are thinner than the $t_{c}$, the two surfaces will come into contact due to the corrugations of the capillary waves, causing the film to break [10], and this theory have been utilized in several studies of layer stability [11,12,13]. Due to computational limitations, molecular simulations of thin layers are commonly carried out in simulation cells with very small interfacial areas, *A_i_*, in the scale of a few nm^2^, where the full extent of the capillary waves cannot manifest. Simulations on *A_i_* as large as 600 nm × 600 nm [14] did not develop capillary waves or other thermal fluctuations large enough to break the films; therefore, other phenomena should arise at this peculiar state. Additionally, other phenomena occur as the thickness of the layer is reduced. Two important properties of the polyethylene layer shift to a lower *T*: the glass transition temperature, *T_g_* [15,16,17,18], and the melting temperature, *T_m_* [19,20], which indicate an effect of the layer thickness on its cohesivity.

In this paper, we investigated nanothin films (<100 nm) of melted polyethylene chains in a vacuum, without impurities or large thermal fluctuations, through molecular dynamics simulations. These simulations were used to predict the limits of mechanical stability over a wide range of *T* values, below the bulk *T_m_*, and up to the limits of thermal stability. We examined interfacial and structural properties (layer and interface thickness, bulk liquid densities, and radius of gyration, *R_g_*, distributions) for thin layers at their specific $t_{c}$ values and compared their interfacial behaviors with those of wider layers.

## 2. Methodology

The liquid–vacuum interface of melted polyethylene chains was studied directly through the simulation of a thin layer of liquid in a vacuum using the molecular dynamics method. The system was expected to behave similarly to ionic liquid films with very low vapor pressure and thermal degradation at high *T* values [21,22]. We simulated layers at *T* values of 373.15, 473.15, 573.15, and 673.15 K, below the bulk *T_m_* of linear polyethylene (403.65 K for a molecular weight of 100.5 kg/mol) [20], beyond the highest *T_g_* reported (253 K) [23,24], up to *T* values near the limit of thermal stability (losses of 5% of the weight at 695.55 K, and the maximum loss begins at 747.45 K) [25,26]. The simulation cell consisted of a parallelepiped with $A_{i}=145\;Å\times 145\;Å$, which was large enough to contain a stretched chain in each lateral direction, had a variable length in the inhomogeneous direction (depending on the number of chains simulated), and contained between 93 and 1568 C_200_ polyethylene chains. Chains of 200 carbon units were chosen as a tradeoff between computation time and chain size; larger chains required a larger *A_i_* to find the *t_c_*. The chosen *A_i_* is not large enough to avoid size effects; based on the results for atomistic systems at a *T* close to the triple point, a simulation cell ~20 times larger the lateral length used in this work is needed to minimize the size effects [8,14]; it is estimated that our calculations for *t_c_* will have underpredictions of ~30% at temperatures close to the shifted *T_m_*.

The initial systems consisted of ordered, stretched, and oriented chains that were parallel to the interfacial surface, with a chain–chain separation of 5 Å in all directions. The initial position of the sites in the configuration were obtained using an in-house code with randomly distributed velocities. The chains were brought to the liquid–vacuum equilibrium slowly over a period of 1 ns, and the mechanical stability was studied using additional simulations in the NVT ensemble (constant number of molecules, rigid volume of simulation cell, and constant average *T*) ensemble, with a timestep of 1 fs. The thermostat used was that of Nosé [27], implemented in the Large-scale Atomic/Molecular Massively Parallel Simulator (LAMMPS) [28].

The chains interacted through the TraPPE potential [29], which is a united atom potential that considers the CH_3_ and CH_2_ functional groups as Lennard–Jones sites of interaction. The non-bonded Lennard–Jones sites interacted through their corresponding potential,

(1) $$U_{LJ}=4\pi \varepsilon_{ij}\left[{\left(\frac{\sigma_{ij}}{r_{ij}}\right)}^{12}-{\left(\frac{\sigma_{ij}}{r_{ij}}\right)}^{6}\right]$$

where *r_ij_* represents the reduced separation between Lennard–Jones sites i and j. *σ_ij_* and *ε_ij_* are parameters dependent on the interaction site; for unlike sites, standard arithmetic ($\sigma_{ij}$) and geometric (*ε_ij_*) combining rules were used. We used a long cutoff radius, $r_{c}=7.5\;\sigma_{CH_{3}-CH_{3}}$, to consider all potential interactions and avoid the use of long-range corrections at the end of the simulations, or approximated corrections during the simulation, which masks the true dynamics of the system [8,14,30]. The use of a short $r_{c}$ with long-range corrections can lead to correct predictions of some liquid–vapor and liquid–vacuum equilibrium properties, but the dynamics of the system are dependent on the $r_{c}$ employed [14,21]. The TraPPE potential for linear alkanes uses harmonic potentials for bond and angle bending and a cosine function on the dihedral angles [31]. The TraPPE potential has been employed to study the structural conformations of single polyethylene chains (C_1000_) [32], semicrystalline polyethylene (C_112_) [33], and melted polyethylene (C_30_–C_150_) [34] and polyethylene blends (C_320_) [35]. The use of the TraPPE potential to study the crystallization process of polyethylene (C_192_) has produced results that are in better agreement with experiments compared to when other potentials are used [36].

Liquid interfaces are dynamic, and under vacuum or surrounded by vapor phases, they breathe; the thickness of the layer and the thickness of each interface expands and contracts in a characteristic periodical behavior. Therefore, to properly characterize the distributions of their structural properties we need to demarcate the different regions of the layer: bulk liquid-like and interfacial regions. A commonly used delimiting point of the interfacial regions is the location in the inhomogeneous direction of the Gibbs dividing surface, which is commonly used in binary and multicomponent phase equilibria (including a solvent) to delimit homogeneous fluid phases (vapor or liquid). In this paper, the Gibbs dividing surface delimits the liquid-like regions from the homogeneous vacuum. For each interface (the free-standing layer has 2 liquid–vacuum interfaces), the Gibbs dividing surface is the point in the interface that divides each interfacial region into two sub-regions (one rich and another poor in solvent), and the criteria for the location of the point is that the sub-region poor in solvent will contain an amount of solvent equal to the amount of solvent “lost” (compared to the bulk phase) in the sub-region rich in solvent. For one-component systems, the only component is considered as the solvent of the system.

To obtain more realistic profiles, we allowed the system to move in the inhomogeneous direction and calculated density profiles every 100 steps. In the end, we averaged the profiles by correcting the positions of the profiles according to the position in the inhomogeneous direction of the center of the layer, which was calculated as the midpoint between the two positions of the Gibbs dividing surface,$\;z_{Gds}$. This allowed us to obtain more realistic profiles of the calculated properties. To obtain the positions of $z_{Gds}$, each of the density profiles (calculated using bins of 0.1 Å) were adjusted to the hyperbolic tangent expression commonly used in liquid–vacuum phase equilibrium studies [21,22]:

(2) $$\rho \left(z\right)=\frac{1}{2}\rho_{l}\;\left[1-tanh\left(\frac{z-z_{Gds}}{d}\right)\right]$$

where $\rho_{l}$ is the average bulk density of the liquid phase. *d* is a measure of the thickness of the interface and describes the length in the inhomogeneous direction, where the density changes from the bulk liquid-like phase to zero in the vacuum. The “10–90” interfacial thickness, $t_{i}=2.1790d$ [37,38], represents the interfacial region in the density profile where the density changes from 10% ($z_{10\%})$ to 90% ($z_{90\%})$ of the average bulk $\rho_{l}$.

**Figure 1:**
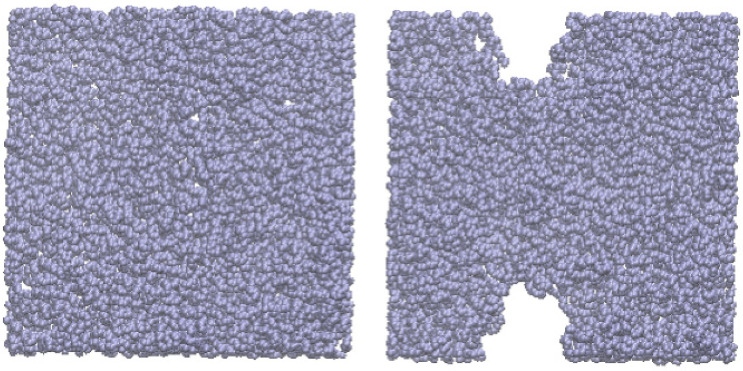
Normal views of the representation of a thin layer (interfaces grow infinitely on the paper plane) of linear polyethylene (C_200_), made of Lennard–Jones sites in equilibrium at *T* = 473 K. The simulation cell used $A_{i}=145\;Å\times 145\;Å$. Thin layers with 102 or more chains (left) were stable up to 0.1 μs, while layers with 101 or fewer chains (right) contracted and developed stable nano-sized holes.

Molecular dynamics simulations of thin fluid layers of melted chains of Lennard–Jones sites representing the behavior of linear polyethylene (C_200_) were performed in an inhomogeneous arrangement to predict the *t_c_* at which the thin layers were at the limit of mechanical stability at a given *T* (Figure 1); narrower layers than those at the *t_c_* shrank, and nano-sized holes formed in the layer (Figure 1). The simulation cell was under periodic boundary conditions and represented a thin layer of infinite *A_i_*.

The search for the minimum number of chains that form stable layers was by trial and error. We started with a stable layer with 200 chains, in which we removed a few chains from the interface and equilibrated the system for 10 ns. If the layer was stable, we continued and eliminated a few chains more, and allowed the system to again reach equilibrium (for additional simulations of 10 ns); otherwise, we used the last stable layer. We repeated this procedure, eliminating a progressively lower number of chains (close to the *t_c_*, we eliminated only 1 chain at each step) until we encountered the thinnest, most stable liquid layer that would not retract itself to form nano-sized holes. Once we found the number of chains required at *t_c_*, we tested the equilibrium and performed additional simulations (100 ns) on the stable thin layer to verify its stability at longer times, because some of the layers became unstable at periods longer than 70 ns; when this happened, we simulated the system with 1 more chain and verified again its stability for 100 ns, until we found the stable system. In a previous publication, we predicted the *t_c_* of atomistic Lennard–Jones free-standing thin layers [8], but those systems did not require long periods to become unstable; they only needed a few ns to become either stable or unstable, and remained stable or unstable for longer periods (100 ns). We focused on investigating systems that maintain mechanical stability and have the smallest possible liquid thickness, which corresponds to the *t_c_* of mechanical stability.

**Figure 2:**
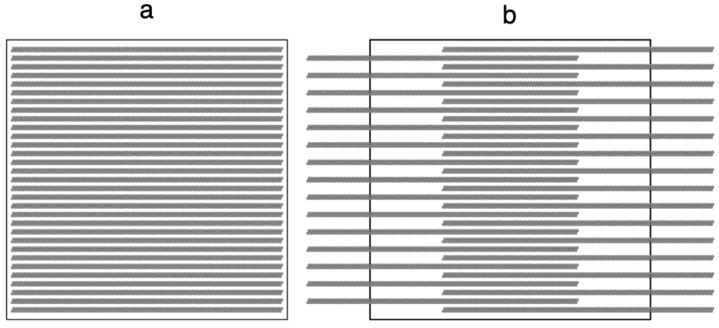
Normal view to the interfacial surface of the representation of uniform (**a**) and non-uniform (**b**) initial configurations of a thin layer of linear polyethylene (C_200_) made of Lennard–Jones sites. The simulation cell used $A_{i}=145\;Å\times 145\;Å$ with periodic boundary conditions (continuous black line).

The equilibration of the system was studied through time profiles of the studied properties, including the total energy of the layer; the system is considered equilibrated because the studied properties reached a plateau, with values oscillating around a constant value. Some properties such as total energy equilibrated faster, but the time of equilibration of the system is established when all properties reached a plateau. To avoid local minima states, additional tests of equilibration were performed through perturbations of *A_i_*; we increased *A_i_* to 5% of its original value in a period of 1 ns and returned to the original *A_i_* value in an additional period of 1 ns. After the perturbation finished, the properties returned to their original values. We tried several initial configurations within our computational restrictions, but most of them failed to produce a stable layer (Figure 2a); the initial configuration that always produced stable layers contained non uniform distributions of chains (Figure 2b). We expected that the tests and perturbations performed, along with the long periods of simulation (100 ns), will produce layers unbiased by the initial configuration.

## 3. Results

Among the properties available for investigation in these very thin layers using molecular dynamics simulations, we studied how the density changes along the two interfaces generated in the liquid–vacuum system, the possibility of adsorption at the interfaces, the width of the homogeneous zone corresponding to the bulk liquid, any distortion in the structure of the chains due to vicinity of the interfaces, and predicted the limits of mechanical stability.

### 3.1. Density Profiles

**Figure 3:**
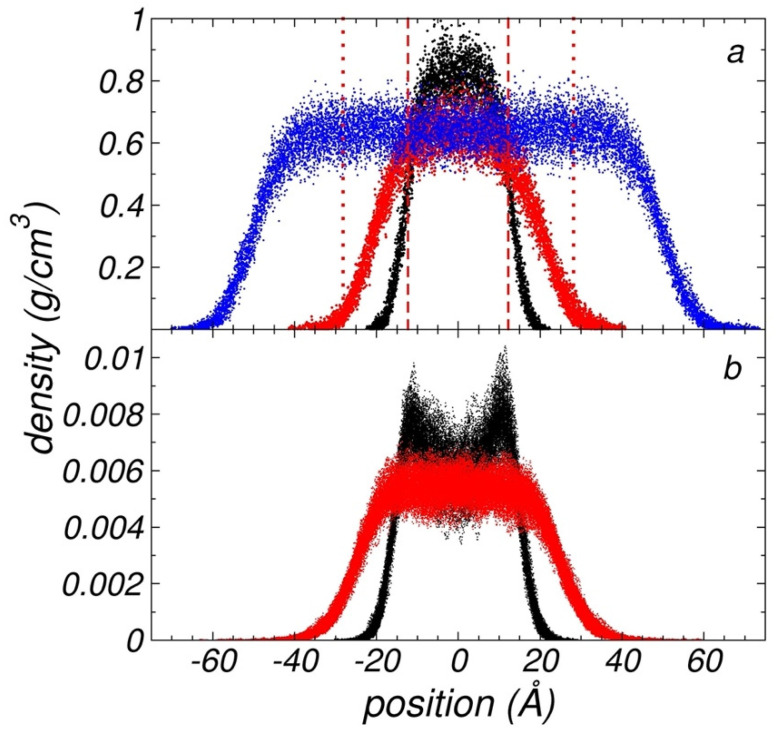
(**a**) Density profiles for the whole system and (**b**) density profiles for only the end CH_3_ groups as a function of the inhomogeneous position within the simulation cell at their corresponding $t_{c}$, for systems of polyethylene chains (C_200_) at *T =* 373.15 K (black) and *T =* 673.15 K (red). Each point represents the average density over 1 ps of simulation. Blue points correspond to a thin layer at 673.15 K with a larger number of chains than the layer at its $t_{c}$. Vertical dashed and dotted red lines represent $z_{90\%}$ and $z_{10\%}$, respectively, for the layer at 673.15 K and at its $t_{c}$.

Figure 3a shows the density profiles of two thin liquid layers of melted polyethylene chains (C_200_) with thicknesses corresponding to their *t_c_* values at 373.15 K and 673.15 K. At 373.15 K, the critical layer consisted of 96 chains, while at 673.15 K, the critical layer needed 116 chains to form a mechanically stable free-standing layer at the same *A_i_*. For comparison, we also show a thin layer at 673.15 K with a thickness wider than the corresponding thin layer at its $t_{C}$. The total density profiles did not exhibit adsorption at the interfaces, probably due to the long length of the chains. Similar density profiles have been reported in the literature for large linear alkanes [39,40,41]. We observed that the system at the lower *T* did not develop a central zone characterized by a flat density, which corresponds to the homogeneous bulk liquid. Meanwhile, the system at the higher *T* had a sufficiently wide thickness to develop a thin bulk liquid-like. When compared with wider thin layers, such as the one shown in Figure 3a, at the same *T* (673.15 K), we found that the average $\rho_{l}$ at the center of the thin layer (−1 Å, 1 Å) at its $t_{c}$ reached the same value as the bulk $\rho_{l}$ of wider layers; this observation was also made for thin layers at the lower *T* of 373.15 K.

End CH_3_ sites are freer to move and associate than backbone CH_2_ sites. We wonder if CH_3_ sites preferred to accumulate in a specific region of the layers, and how such an accumulation affects the properties of the layers. Density profiles of only the end CH_3_ sites of the polyethylene chains are shown in Figure 3b. Density profiles for backbone CH_2_ sites should be very similar to the total profiles, because they represent 99.5% of the total sites. At the higher *T* of 673.15 K, the CH_3_ profiles showed the same trend as the density profiles of the whole system, the only difference being that the CH_3_ profiles reached positions farther away from the bulk liquid (magnified in Figure 3b due to the scale used in the *y*-axis). At the lower *T* of 373.15 K, the density profile showed two peaks of adsorption at the interfaces, which likely indicates that, at this *T*, the system was all interfacial, the system could be considered as two united interfaces, and the adsorption at the interfaces was the result of low mobility of the CH_3_ sites at this lower *T*. The peaks showed the system’s maxima at regions outside the bulk liquid-like regions, where some CH_2_ sites are present, and probably indicate that CH_3_ sites are phobic to the nature of the bulk liquid-like region. The two peaks of density reached values that represent only 1% of the total bulk liquid-like density, even though they are 0.5% of the total number of sites; therefore, its effect on other properties should also be very limited. The valley between the interfaces likely indicates a barrier for the CH_3_ sites exchange in this region; crossing from one interfacial peak to the other may require more thermal energy than is available at this *T*.

### 3.2. Bulk Liquid Density

**Figure 4:**
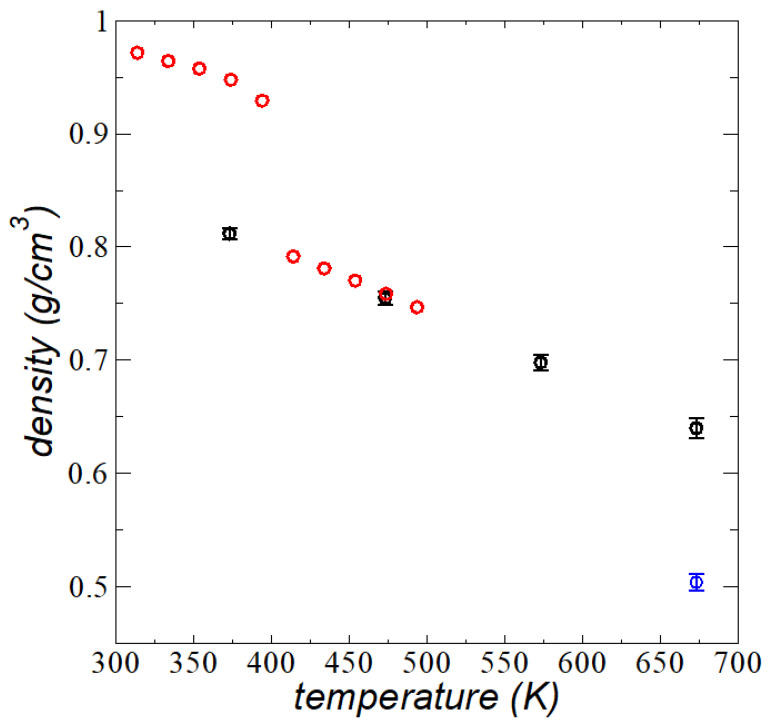
Bulk $\rho_{l}$ of thin liquid layers of melted polyethylene chains (C_200_) in a liquid–vacuum equilibrium as a function of *T*. Black circles represent the results of this work using simulations. The blue circle represents a system simulated at a smaller $r_{c}$ ($2.5\;\sigma_{CH_{3}-CH_{3}}$). Error bars represent the standard deviation of the data. Red circles represent experimental results of Sato et al. for linear polyethylene at 0.1 MPa [Adapted from Ref. [42] with permission from Elsevier under License Number 5163721342541 (7 October 2021)].

By using systems with 288 C_200_ chains and the same $A_{i}$, which form wider thin layers than those at their *t_c_* (more than double the number of chains), we estimated the average bulk $\rho_{l}$ and its standard deviations, which are shown in Figure 4 as a function of *T*. Experimental results for the $\rho_{l}$ of linear polyethylene at 0.1 MPa are also shown in Figure 4 [42]. Ideally, we should have compared our results with measurements in a vacuum, but due to the lack of experiments in vacuum or ultravacuum conditions, we compared our results to the available experimental data at the lowest reported pressure (0.1 MPa), which we expected would be close to the measurements at vacuum conditions. The results obtained from the simulations successfully quantitatively reproduced the available experimental results and their trends at *T* in the range of 473.15–673.15 K.

Between 394 and 414.1 K, the experimental data had a discontinuity, which has been attributed to the transition to molten states; such a transition was not observed in the simulation result at 373.15 K, which followed the trend observed in this work at higher *T* values. The simulated results were obtained after 100 ns of equilibration of the layers, but we observed that, at a *T* in the range of 473.15–673.15 K, the layers equilibrated in shorter periods (10 ns), while the system at 373.15 K required the full 100 ns to equilibrate, which indicates that the system at this *T* was likely near or at the region of metastability. It is also well known that the thickness of polymer layers affects the *T_g_* [15,16,17,18] and *T_m_* [19,20], so the observed trend in this work may indicate that the *T_m_* at nanothin thicknesses was lower than the *T* reported in the experimental work at bulk liquid conditions (403.65 K for a molecular weight of 100.5 kg/mol) [19,20]. Previously reported simulations using the TraPPE potential have quantitively agreed with the experimental *T_m_* (using C_50_ chains) [43] and *T_g_* (using C_192_ chains) [36] bulk values.

The system is not expected to crystallize under simulation, because bulk simulations studying the specific heat of crystalizing melts have shown that crystallization starts at *T* values below 350 K, with a maximum in the specific heat at 325 K when the cooling rate is of 0.05 K/ns and starts to crystalize below 325 K for a heating rate of 0.5 K/ns [36]. To our knowledge, there are no reported simulations at lower heating rates (probably due to computational restrictions), which will increase the crystallization temperatures.

We also investigated the effect of a smaller ($r_{c}\cdot (2.5\;\sigma_{CH_{2}-CH_{2}}$), which is commonly employed in simulations as a tradeoff due to the computational restrictions of many large systems; the system becomes less cohesive due to a large number of unaccounted interactions, and the $\rho_{l}$ reduces (thickness becomes larger) by up to 22% of the value predicted using the full potential.

### 3.3. Radius of Gyration at the Interfaces

**Figure 5:**
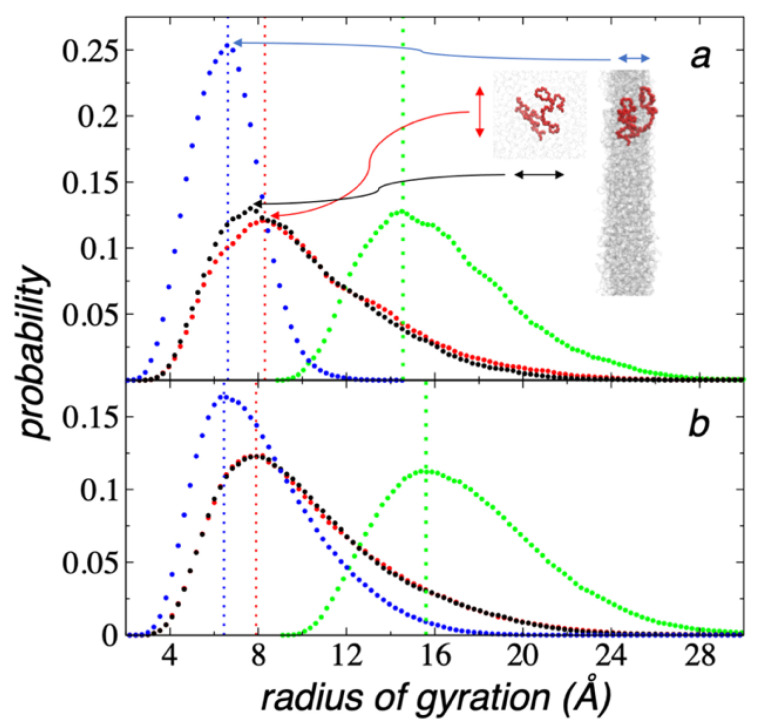
Histograms of the *R_g_* of thin liquid layers of melted polyethylene chains (C_200_) at their $t_{c}$ in a liquid–vacuum equilibrium at (**a**) 373.15 K and (**b**) 673.15 K. Black and red points represent the components in the lateral directions to the interfacial surface, blue points represent components normal to the interfacial surface, and green points represent the total values of *R_g_*. Vertical lines represent the mode of the distributions. Snapshots in (**a**) represent normal and lateral views of a chain with *R_g_* values equal to the mode values of its correspondent distribution (highlighted with red spheres). The rest of the chains are represented with gray lines.

The interfaces of the thin layers at their $t_{c}$ were characterized by histograms (calculated using 200 bins) of the *R_g_*. Guided by the results of the density profiles at these critical conditions, the layers could be regarded as the union of two interfaces, and all chains were considered in the histograms. The histograms for the components, lateral and normal to the interfacial surface, and the total value of *R_g_* for the critical thin layers at 373.15 and 673.15 K are plotted in Figure 5a,b, respectively. The histograms of *R_g_* could be characterized as right-skewed distributions with long tails. The distributions of the lateral components of *R_g_* overlapped each other at 673.15 K, while at 373.15 K, there was a small mismatch close to the top of the distribution, probably indicating that the system was not fully equilibrated even after 100 ns of simulation. A comparison of the lateral distributions of *R_g_* at both *T* values revealed an overlapping of the distributions. The comparison between the distributions of *R_g_* in the normal direction at both *T* values showed a more physically intuitive behavior; the mode of the two distributions were the same, but a shorter peak and a longer tail were observed at the larger *T* (673.15 K), which resulted in a larger average value at 673.15 K. The mode in the normal direction were lower by approximately 1.7 Å than those observed in the tangential direction. The distributions of the total *R_g_* showed small differences; the mode and mean values grew by around 1 Å when the *T* changed from 373.15 to 673.15 K. Similar changes in the total *R_g_* have been reported in atomistic simulations of bulk linear polyethylene [44]. The distributions obtained in this work can be interpreted as a deformation of the chain coils in the normal direction to the interfacial surface. Such a deformation increased as the *T* values were lowered. The deformation of the chains in the normal direction as a function of the film thickness has been reported previously from experiments of thin film polymers expanding freely on the surface of water [45], and also observed in the free surface of supported polymers [46]. The lateral and normal snapshots of a chain coil with *R_g_* values corresponding to the modes of their corresponding distributions (373.15 K) showed that chains are contained in well-defined interfaces, and they cover only part of the layer in the normal direction, even though they had space within the layer to expand in that direction; therefore, the observed deformations in the normal direction are probably due to the interaction with the closest interface.

**Figure 6:**
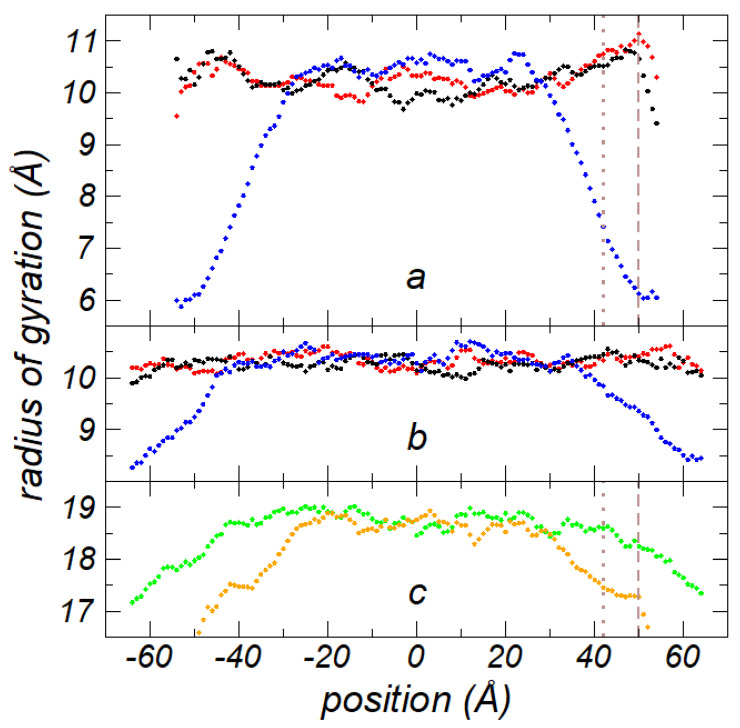
*R_g_* profiles as a function of the inhomogeneous position within the simulation cell at thickness layers wider than the corresponding *t_c_* for systems of 288 polyethylene chains (C_200_) at *T =* 673.15 K, simulated using a $r_{c}$ of (**a**) $7.5\;\sigma_{CH_{3}-CH_{3}}$ and (**b**) $2.5\;\sigma_{CH_{3}-CH_{3}}$. Black and red points represent the lateral *R_g_*, while blue points represent the normal *R_g_*. (**c**) Total *R_g_* profiles for systems simulated using $r_{c}=7.5\;\sigma_{CH_{3}-CH_{3}}$ (orange) and $r_{c}=2.5\;\sigma_{CH_{3}-CH_{3}}$ (green). Vertical dashed and dotted lines in (**a**) and (**c**) represent $z_{Gds}$ and $z_{90\%}$, respectively.

To compare the values of *R_g_* obtained at the $t_{c}$ with those at bulk liquid phases, we computed the *R_g_* profiles for nanothin layers containing 288 C_200_ chains, which represented layers with more than double the number of chains at the $t_{c}$ at the same *T*. The profiles for the components of *R_g_* and its total value at 673.15 K and at its $t_{c}$ are shown in Figure 6a,c, respectively. The profiles were averaged over the total period of properties estimation (10 ns). Compared to the density profiles, the *R_g_* profiles were not uniform, because the *R_g_* calculation produced only one point per chain at its center of mass, and the density profiles produced 200 points (corresponding to the CH_2_ and CH_3_ sites). Only bins in the profiles with more than 1% of the average number of chains in the bulk $\rho_{l}$ were plotted. The position in the normal direction to the surface interface, at which the *R_g_* value was assigned, corresponded to the center of mass of the chain. The lateral components remained constant along the axis in the normal direction, while the average magnitude of the normal component was constantly reduced in the interfacial region. The reduction in the average normal *R_g_* reached around 4.4 Å at the outermost layer of the interface plotted. Molecular simulations at the free surface of supported polymer films have revealed a similar behavior: deformations of the chain coil in the normal direction at interfaces with very low sensitivity to *T* and insensitivity to the bulk $\rho_{l}$ [46]. The profile for the total *R_g_* showed the same behavior as the component in the lateral directions, with an average value in the center of the layer of 18.67 ± 0.14 Å.

We compared the *R_g_* profiles to those obtained using a smaller $r_{c}$ of $2.5\;\sigma_{CH_{3}-CH_{3}}$, and the components and total value of *R_g_* are shown in Figure 6b,c, respectively. The profiles behaved very similarly to those using a longer *r_c_*, and they were wider due to the lower cohesivity and density of the system (Figure 4). The total *R_g_* showed a slightly higher average value in the center of the layer of 18.77 ± 0.15 Å, and the maximum deformation of the chains in the normal direction was approximately only 2.1 Å. The insensitivity of the total *R_g_* to the bulk $\rho_{l}$ of both systems at different *r_c_* values indicates that interactions within each chain (intrachain) were probably insensitive to *r_c_* (the same *r_c_* was used for inter- and intra-chain interactions). Interactions between different chains are sensitive to *r_c_* and dictated the density of the layer. The smaller variation of the deformation in the normal direction using a smaller *r_c_* was likely the result of unaccounted bulk and interfacial chain–chain interactions.

**Figure 7:**
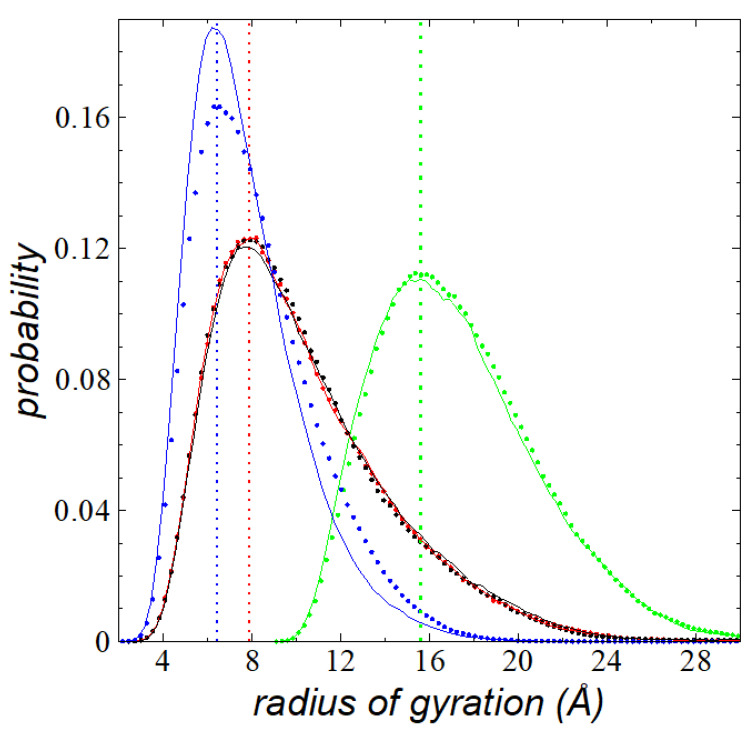
Histograms of *R_g_* of thin liquid layers of melted polyethylene chains (C_200_) in a liquid–vacuum equilibrium at their t*_c_* at 673.15 K. Black and red points represent the components in the lateral directions to the interfacial surface, blue points represent the normal direction, and green points represent the total values. Continuous lines represent the histograms for the interfacial chains of a wider thin layer than the layer at its t*_c_* containing 288 chains; the color code in continuous lines is the same as the lines of points. Vertical lines represent the mode of the distributions.

The effect of the layer thickness on interfacial *R_g_* distributions was evaluated through a comparison of the distributions of *R_g_* at two different thicknesses at the same *T*. In Figure 7, the distributions of *R_g_* for the total value and its components are shown for thicknesses at its *t_c_* (116 chains) and for a thicker layer with 288 chains, both at a *T* of 673.15 K. For the thicker layer, we accounted for the chains located not only at the interface defined as $t_{i}$, but also deeper in the bulk liquid, where the normal components of *R_g_* started to deform: (−∞, −28 Å) for the left interface and (28 Å, ∞) for the right interface. The distributions of the total value and the components in the lateral directions did not change, but the distribution in the normal direction increased in magnitude close to the region of the mode and decreased the number of chains with higher values. This can be interpreted as a slight increment in the number of molecules that were highly deformed in this region, which was the result of the increment of the thickness of the layer. This effect would likely disappear at a larger thickness once the cohesivity of the liquid layer reached a saturation value.

### 3.4. Critical Thickness

**Figure 8:**
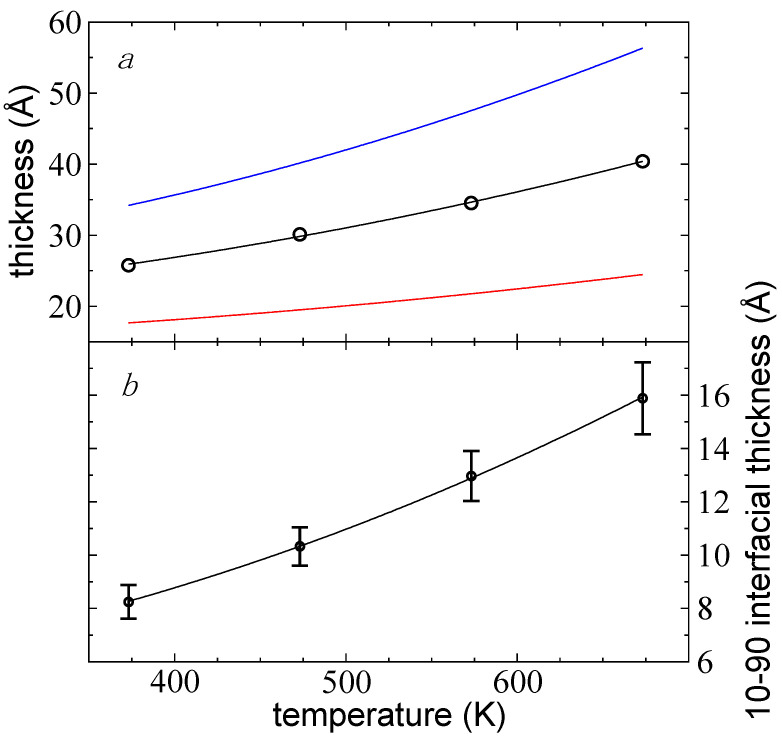
(**a**) $t_{Gds}$ (black circles) of thin liquid layers of melted polyethylene chains (C_200_) in a liquid–vacuum equilibrium as a function of *T*. Standard deviations are smaller than the symbols. The black line represents the adjustment to a quadratic function on *T*. (**b**) $t_{i}$ (black circles) as a function of *T*. The solid line represents the adjustment to a quadratic function on *T*. The bars represent standard deviations of the measurements. Red and blue lines in (**a**) represent $t_{Gds}-t_{i}$ and $t_{Gds}+t_{i}$, respectively.

A measurement of the thickness of thin layers at their limit of mechanical stability was obtained by adjusting the density profiles, averaged every 100 fs using Equation (2). From these adjustments, we obtained 100,000 values (10 ns of properties evaluation) for two parameters that characterized the components of the system (interfaces and “bulk” liquid zone): $t_{Gds}$ and $t_{i}$. $t_{Gds}$ is the separation in the normal direction between the $z_{Gds}$ values of the interfaces. $t_{Gds}$ and $t_{i}$ increased as *T* increased and are shown in Figure 8a,b, respectively. The increase in $t_{Gds}$ was not only due to the change of density due to increasing *T*, but additional chains were also needed to mechanically stabilize the thin layer. The data obtained for both properties showed normal distributions. The standard deviations for $t_{i}$ are plotted in Figure 8b as the errors in the measurements (for $t_{Gds}$; the deviations are smaller than the symbols); the standard deviations also increased with *T*. The sets of data for both properties adjusted well to empiric quadratic expressions on *T*. At the largest *T* studied, where the interfacial thickness was at a minimum, the characteristic values were 25.76 Å ($t_{Gds}$) and 8.23 Å ($t_{i}$). If comparisons were made with experimental measurements, it would likely be difficult to determine experimentally where the interfaces ended due to the breathing of the interfaces. Depending on the sensitivity of the experimental technique, different values could be reported. Based on this probable experimental uncertainty, we proposed a criterion for the prediction of $t_{C}$ in a range of separations between the two interfaces, at surfaces at each interface located between 10% ($t_{Gds}+t_{i}$) and 90% of the bulk $\rho_{l}$ ($t_{Gds}-t_{i}$). The experimental detection of layers with less than 10% of the total $\rho_{l}$ would likely be very difficult to achieve due to the dynamics of the interfaces (breathing). At 373.15 K, $t_{c}$ would be in the range of 17.67–34.20 Å. Both thicknesses, $t_{Gds}-t_{i}$ and $t_{Gds}+t_{i}$, are represented in Figure 3a by the vertical limits for the thin layer at its $t_{c}$ at 673.15 K. The proposed criterion was based on the importance of long-range dispersion forces (van der Waals) at the interfaces, which create disjoining pressures between the interfaces and were more influential as the thickness of the layers decreased [11,47,48,49,50]. The range of values for $t_{c}$ at 373.15 K represented between 4.5 and 8.7 times the value of the Lennard–Jones parameter $\sigma_{CH_{3}-CH_{3}}$. The minimum in the Lennard–Jones potential is at $2^{1/6}\sigma \;\sim \;1.12\sigma$, and the separation between contiguous sites is around this value. Therefore, at 373.15 K, each interface of the bulk layer could be viewed as two monolayers, with the outermost monolayer moving freely into the vacuum, up to distances corresponding to two monolayers ($\sim \;2.24\sigma_{CH_{3}-CH_{3}}$). At 673.15 K, $t_{c}$ was in the range of 6.2–14.3 times $\sigma_{CH_{3}-CH_{3}}$ and could be viewed as interfaces consisting of three monolayers, with the outermost monolayer moving into the vacuum up to distances corresponding to four monolayers.

## 4. Conclusions

Molecular dynamics simulations under vacuum conditions predicted $t_{c}$ values for free-standing melted and linear polyethylene C_200_ chains as small as 1.77–3.42 nm at 373.15 K (depending on where the thickness was measured) and as high as 2.45–5.63 nm at *T* values near the limit of thermal stability (673.15 K). The required thickness to maintain mechanical stability increased with the square of *T*. The $t_{c}$ not only increased due to the reduction in the layer density as *T* increased, but additional chains were also needed to ensure mechanical stability; approximately 44 chains per 100 nm^2^ at 373.15 K and 55 chains at 673.15 K. At the $t_{c}$, the system was mostly formed by two united interfaces with interfacial thicknesses between 0.83 nm at 373.15 K and 1.59 nm near the limit of thermal stability.

The layer thickness did not affect interfacial behavior, because the interfaces at the $t_{c}$ developed the same average density profiles and interfacial thicknesses as wider layers. There was not a proper bulk liquid at the $t_{c}$ as there was in wider layers, but at the center of the layer (−1 Å, 1 Å), the density reached values equal to those found in the average bulk liquid of wider layers, which reproduced experimental values previously reported. The liquid density of the layer at 373.15 K followed the trend observed in this work at higher values of *T*, indicating that the melting point of these nanothin layers was affected by the thickness of the layer and should be below 373.15 K.

The distributions of the *R_g_* showed the independence of the interfacial dynamics of the chains with the layer thickness, and they also demonstrated a deformation in the normal direction of the chain coils, probably due to the high attraction between the two conjoined interfaces. The mode of the right-skewed distributions of the *R_g_* was independent of *T*; as *T* increased, the right tail of the distribution also increased, producing larger average values.

The studied systems did not show capillary waves large enough to be the factor that make the layers to break. The studied structural and interfacial properties did not show an enhancement (or reduction) as the thickness of the layer was reduced, not even when they reached its critical thickness; therefore, in future work, we will investigate the role of other properties as the interfacial forces on the mechanical stability at the critical thickness.

## Data Availability

The data presented in this study are available on request from the corresponding author.
